# Distinct Neurogenomic States in Basal Ganglia Subregions Relate Differently to Singing Behavior in Songbirds

**DOI:** 10.1371/journal.pcbi.1002773

**Published:** 2012-11-08

**Authors:** Austin T. Hilliard, Julie E. Miller, Steve Horvath, Stephanie A. White

**Affiliations:** 1 Department of Integrative Biology and Physiology, University of California Los Angeles, Los Angeles, California, United States of America; 2 Interdepartmental Program in Neuroscience, University of California Los Angeles, Los Angeles, California, United States of America; 3 Departments of Human Genetics and Biostatistics, University of California Los Angeles, Los Angeles, California, United States of America; 4 Interdepartmental Program in Molecular Cellular & Integrative Physiology, University of California Los Angeles, Los Angeles, California, United States of America; University of Oxford, United Kingdom

## Abstract

Both avian and mammalian basal ganglia are involved in voluntary motor control. In birds, such movements include hopping, perching and flying. Two organizational features that distinguish the songbird basal ganglia are that striatal and pallidal neurons are intermingled, and that neurons dedicated to vocal-motor function are clustered together in a dense cell group known as area X that sits within the surrounding striato-pallidum. This specification allowed us to perform molecular profiling of two striato-pallidal subregions, comparing transcriptional patterns in tissue dedicated to vocal-motor function (area X) to those in tissue that contains similar cell types but supports non-vocal behaviors: the striato-pallidum ventral to area X (VSP), our focus here. Since any behavior is likely underpinned by the coordinated actions of many molecules, we constructed gene co-expression networks from microarray data to study large-scale transcriptional patterns in both subregions. Our goal was to investigate any relationship between VSP network structure and singing and identify gene co-expression groups, or modules, found in the VSP but not area X. We observed mild, but surprising, relationships between VSP modules and song spectral features, and found a group of four VSP modules that were highly specific to the region. These modules were unrelated to singing, but were composed of genes involved in many of the same biological processes as those we previously observed in area X-specific singing-related modules. The VSP-specific modules were also enriched for processes disrupted in Parkinson's and Huntington's Diseases. Our results suggest that the activation/inhibition of a single pathway is not sufficient to functionally specify area X versus the VSP and support the notion that molecular processes are not in and of themselves specialized for behavior. Instead, unique interactions between molecular pathways create functional specificity in particular brain regions during distinct behavioral states.

## Author Summary

Understanding how gene transcription relates to behavior is challenging. Learned vocal-motor behavior is a complex trait that represents the output of multiple converging genes, pathways, and patterns of neural activity. Here, we applied a systems analytical approach to determine how thousands of genes change their expression levels simultaneously in a region of the vertebrate brain important for vocal-motor function, the basal ganglia, during a specific vocal-motor behavior, singing. We used the zebra finch species of songbird based on similarities between song learning/production and speech, and because they possess a set of brain subregions dedicated to singing. Microarrays were used to measure gene expression levels in one such song-dedicated region and in an adjacent motor area that is not thought to play a role in vocal function. This allowed us to address the question of whether distinct gene co-expression patterns could be found in each area. We found that each area contained unique patterns of transcriptional co-activity, but there were also unexpected overlaps. We conclude that the particular behaviors (singing versus non-vocal behaviors) supported by these subregions depend on the particular sets of interactions between molecular pathways that occur in each subregion.

## Introduction

**Figure 1:**
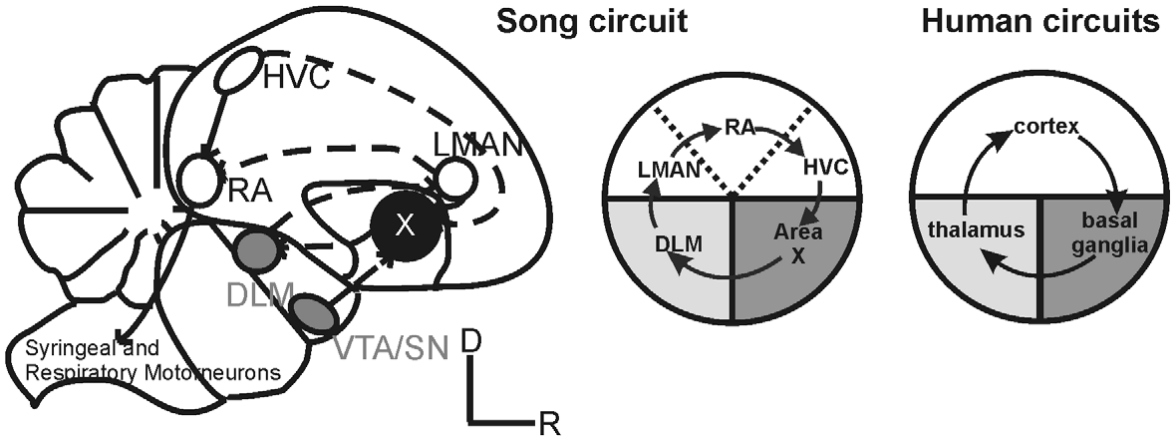
Basal ganglia sub-regions selected for comparison. **Left -** Composite sagittal view of the songbird brain indicates song control nuclei and their connections. Auditory input (not shown) enters the song circuit at HVC (acronym used as proper name), the neurons of which contribute to both the posterior vocal motor pathway (plain arrows) and the anterior forebrain pathway (stippled arrows). The latter includes area X, and rejoins the posterior pathway via projections from the lateral magnocellular nucleus of the anterior nidopallium (LMAN) to the robust nucleus of the arcopallium (RA). Middle and right) Schematic comparison of avian and human cortico-basal ganglia loops [32]. Song-specialized sub-regions are embedded within similar brain areas in the human brain. Cortex is shown in white, basal ganglia in dark grey and thalamus in light grey.

The basal ganglia are a network of subcortical nuclei involved in diverse types of motor function, ranging from simple reflexive habits to deliberated, goal-directed actions. The striatum, the largest basal ganglia nucleus, contains anatomically and functionally separate sub-regions known to mediate distinct forms of motor control and learning in mammals, and investigation of the molecular mechanisms underlying striatal control of learned motor behaviors is an active research area [1]. What has been traditionally called the ‘striatum’ of songbirds such as the zebra finch corresponds to the mammalian striatum, but also contains pallidal neuron types and thus is more appropriately termed the striato-pallidum. As in mammals, the songbird striato-pallidum is active during voluntary movements [2]. In contrast to mammals, the neurons that are dedicated to the learning and production of specific vocal-motor sequences are grouped together in a dense nucleus. This sub-region is called area X and is part of brain-wide circuitry dedicated to song (**Figure 1**) [2]. This unique specification allowed us to compare transcriptional patterns in tissue dedicated to vocal-motor function (area X) to those in tissue that is composed of similar cellular phenotypes but supports non-vocal motor behaviors, such as hopping and wing beating; the striato-pallidum ventral to area X (VSP). Some non-vocal behaviors, such as courtship dances, co-occur with singing [3], [4]. Comparison of immediate early gene expression between individual cases in which a bird moved but did not sing, versus one that sang but did not make many other movements, indicate activation of the VSP for the former and of area X for the latter [5]. Because of the striking functional contrast between the VSP and area X, we expected to observe different gene expression patterns in these 2 regions during singing. We also hypothesized that VSP gene expression patterns would bear a less significant relationship to singing than those in area X.

Since any behavior is likely to be supported by the orchestrated actions of many molecules, as opposed to a single gene or pathway, we used gene microarrays to collect expression data simultaneously from many thousands of genes in both area X and VSP of 27 adult zebra finches. To identify groups of genes with similar mRNA expression profiles across birds, we performed weighted gene co-expression network analysis (WGCNA) [6], a technique that has uncovered patterns of gene co-activity correlated to biological traits and corresponding to functional pathways in multiple species [7], [8].

In a previous study, we applied WGCNA to microarray data arising only from area X. This resulted in a large network of ∼20,000 gene probes from which we were able to identify groups of genes, known as modules, whose expression variability across birds was highly correlated to singing and vocal variability [9]. The co-expression relationships of genes in these area X “song modules” were not preserved in the VSP data, and overall we determined that the more strongly related to singing a given area X module was, the less preserved it was in the VSP. These insights relied on the use of powerful module preservation statistics that evaluated whether modules of a reference network (the large area X network) were preserved in the test dataset (VSP). An advantage of these preservation statistics is that they allowed us to rigorously argue that certain modules were not preserved since they did not make use of module assignments in the test data set [10]. But a limitation of our previous study was that we never constructed modules in the VSP. We were thus precluded from identifying any VSP-specific modules, either by computing statistics of VSP module preservation in area X, or by directly comparing the gene composition of modules in each subregion. Our prior results led us to the hypothesis that a VSP co-expression network would share some similarities with area X, but would also exhibit its own unique patterns of transcriptional co-activity. Here we tested this hypothesis by reversing the roles of the area X and VSP data sets: identifying co-expression modules in the VSP and then evaluating their preservation in area X. This approach allowed us to distinguish between different hierarchical transcriptional profiles in adjacent brain regions that control different behaviors but are composed of the same building blocks.

Our hypotheses were largely confirmed; a group of 4 VSP modules was well preserved in the new area X network while other modules were highly specific to the VSP, and we did not detect the same strong correlations to the amount of singing as we did in area X. Interestingly, however, we did observe patterns of relatively weak correlations to song features across the VSP modules. These patterns were most distinct with regard to song features that were not highlighted in our previous area X findings, implying that molecular processes outside of proper song control regions may contribute, at least indirectly, to vocal-motor performance. In addition, VSP-specific modules were enriched for processes disrupted in Parkinson's Disease (PD) and Huntington's Disease (HD).

Because we could directly compare module assignments in the two networks, we were able to determine that genes in two VSP-specific modules were split across multiple area X modules into functionally distinct groups. This strongly supports the notion that single genes and molecular pathways are not in and of themselves specialized for systems-level neural functions or behavior, but instead unique hierarchical patterns of interactions between pathways combine to create functional specificity in particular brain regions under certain conditions.

## Methods

### Animals and behavior

Animal use was in accordance with NIH guidelines for experiments involving vertebrate animals and approved by the University of California at Los Angeles Chancellor's Institutional Animal Care & Use Committee. Animals and song recording and analysis procedures were identical to those described in [9].

### Tissue collection, RNA isolation, and microarrays

Tissue collection, RNA isolation procedures, and details about the microarrays are described in [9]. Area X and VSP datasets (raw and processed data) are available at the Gene Expression Omnibus (www.ncbi.nlm.nih.gov/geo) under accession number GSE34819. Scanned microarray images were analyzed with Feature Extraction Software 9.5.3.1 (Agilent, protocol GE1-v5_95 and Grid: 019785_D_F_20080327) to obtain background subtracted and spatially detrended Processed Signal intensities, which were the input to further data pre-processing.

### Data pre-processing

All data pre-processing and co-expression network analysis was done in the freely available statistical software R (www.r-project.org) and the WGCNA R library [11]. R functions written by ATH for array pre-processing are available at https://www.ibp.ucla.edu/research/white/code.html. Removal of outlier probes (including those with sub-background expression levels) and outlier samples was performed as described in the supplemental information of our previous area X-based study [9], with the following additions: In the previous analysis, ∼1/2 of the probes on the microarray (n = 20,104) were used for WGCNA, and in most cases there were multiple probes for the same gene in the network, e.g. there were 12 probes for *FOXP2*. A large portion of the network was also made up of probes for genes whose identity was unknown. In order to streamline the present VSP-based study and ease interpretation of the results, unannotated probes were removed, and one representative probe was selected for each gene using the collapseRows() function, leaving 11,482 genes. By default, representative probes are chosen as those with the highest average expression values across samples since they tend to yield the most reproducible results [12].

### Essential network terminology

**Figure 2:**
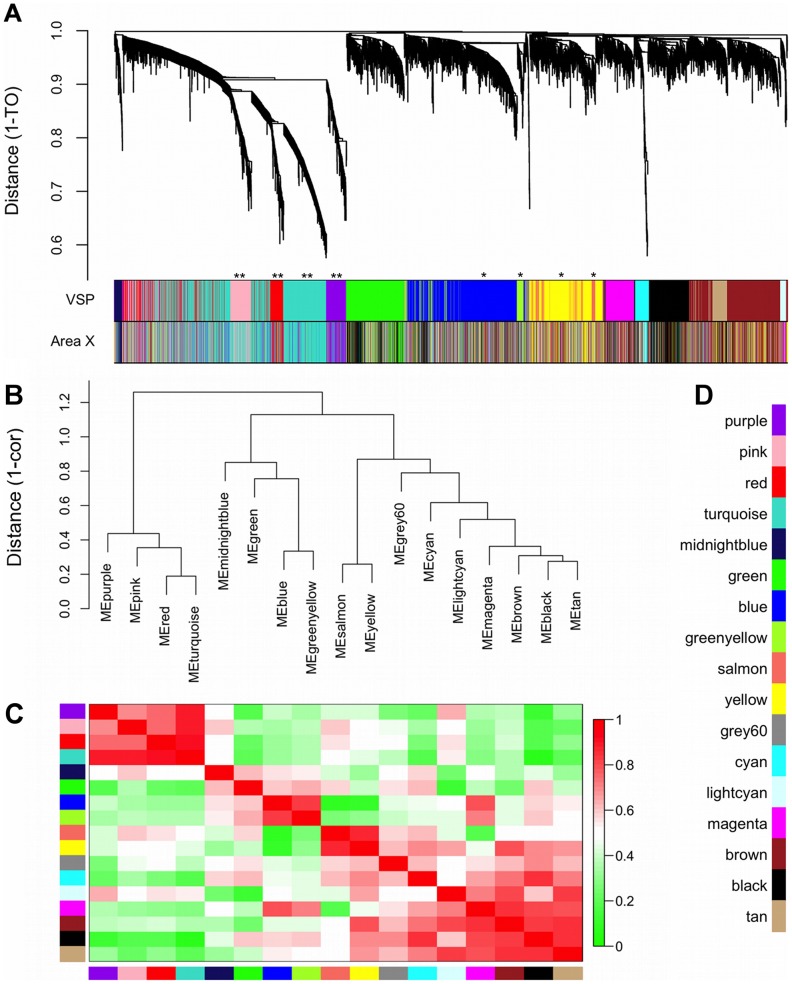
Gene co-expression modules and meta-modules. (**A**) Dendrogram shows the results of hierarchically clustering genes in the VSP. “Leaves” along “branches” represent gene probes. The y-axis represents network distance as determined by 1 – topological overlap (TO), where values closer to 0 indicate greater similarity of probe expression profiles across samples. Color blocks below denote modules in the VSP (top) and area X (bottom) that probes were assigned to in each region. Area X module colors were set to match the VSP colors as closely as possible, thus blocks of color that match in the 2 bands indicate VSP module preservation in area X (e.g. **pink, red, turquoise, purple). *Unpreserved modules: blue, green-yellow, yellow, and salmon. (**B**) Dendrogram shows the results of hierarchically clustering VSP module eigengenes (MEs) to examine higher-order relationships between the modules. “Leaves” along “branches” represent MEs. The y-axis represents network distance as determined by 1 – correlation, where values closer to 0 indicate greater similarity between main sources of expression perturbation in the modules. (**C**) Heatmap representing correlations between VSP MEs. Each row/column represent a ME, indicated by module color blocks on the x- and y-axes. Dark red indicates strong positive correlation, dark green indicates strong negative correlation, and white indicates no correlation, as indicated in color scale bar. Cells on the diagonal are dark red because each ME correlates perfectly with itself. (**D**) Color key for all VSP modules. The same colors are used throughout subsequent figures.

Co-expression networks were constructed by hierarchically clustering genes based on topological overlap (TO), a biologically meaningful measure of node interconnectedness that compares patterns of gene connection strengths to quantify similarity in the context of the entire network. Other similarity measures such as correlation or Euclidean distance only consider each gene-gene pair in isolation. Thus, TO is effective at filtering spurious or isolated connections, and probes with high TO have increased chances of involvement in the same biological pathways [13]. Modules were defined as branches of the dendrogram obtained from clustering, and labeled by arbitrary colors underneath the dendrogram (**Figure 2A**). By convention, genes that were not assigned to any module were considered background, i.e. not correlated with genes in the network, and labeled by the color grey. To study module composition, module “eigengenes” (MEs) were defined as the 1^st^ principal component of each module, effectively summarizing the expression variability within modules. MEs were used to quantitatively relate gene co-expression patterns to phenotypic traits and construct ME correlation networks to study higher-order relationships among the modules. Module membership (kME; aka eigengene-based connectivity) was defined as a gene's correlation to the ME, thus quantifying the extent to which its expression profile conformed to the largest source of variability within the module. Intramodular connectivity (kIN) was defined as the sum of a gene's network connections with other module members.

### Weighted gene co-expression network analysis (WGCNA)

WGCNA was performed as described in [9], using functions in the R WGCNA library [11], with an additional preliminary filtering step inspired by the procedure used in [14]. Briefly, instead of analyzing a network of all 11,482 genes left after pre-processing, an iterative filtering process was performed to enrich the final network with modules composed only of the most densely interconnected genes. The soft threshold for constructing a signed weighted correlation network (*β* = 14) was determined with the scale-free topology criterion applied to all 11,482 genes. A preliminary network was constructed using default module definition (dynamic tree-cutting) settings, except for a smaller minimum module size of n = 10 genes [15]. The average TO within each module was defined as the module density, which was then compared to the density of 10,000 pseudo-modules of the same size that were generated by randomly selecting genes from the network. A p-value for the density of each module was defined as the number of pseudo-module densities greater than the actual density, divided by 10,000. Genes in modules with p>0.01 and grey background genes were removed. The network was rebuilt with the remaining genes, and the process was iterated until all modules passed the density test and there were no more grey genes, leaving 5,368 genes in the final VSP network. The area X network was constructed with the same 5,368 genes, using the same WGCNA parameter values as in the VSP. No additional filtering was performed in the area X network since it was purposely constructed with reference to the VSP.

To confirm the efficacy of the additional filtering for ensuring VSP module robustness, we computed module quality statistics using the WGCNA function modulePreservation() [10], [11]. Briefly, typical module preservation statistics were used to evaluate the preservation of VSP modules in test networks created by randomly permuting the actual gene module assignments. In this type of comparison, preservation statistics are interpreted as indicators of module density (how tight the interconnections among the genes in a module are) and separability (how distinct modules are from others in the network), i.e. module quality. By averaging preservation statistics across many permutations of the original data, module quality statistics are indicative of module robustness and reproducibility. Z_summary_ scores >10 are interpreted as strong evidence of densely interconnected, distinct, reproducible modules, and scores for the VSP modules ranged from 13.2 (green-yellow) to 77.9 (turquoise; **Figure S1**).

### Comparison to area X

**Figure 3:**
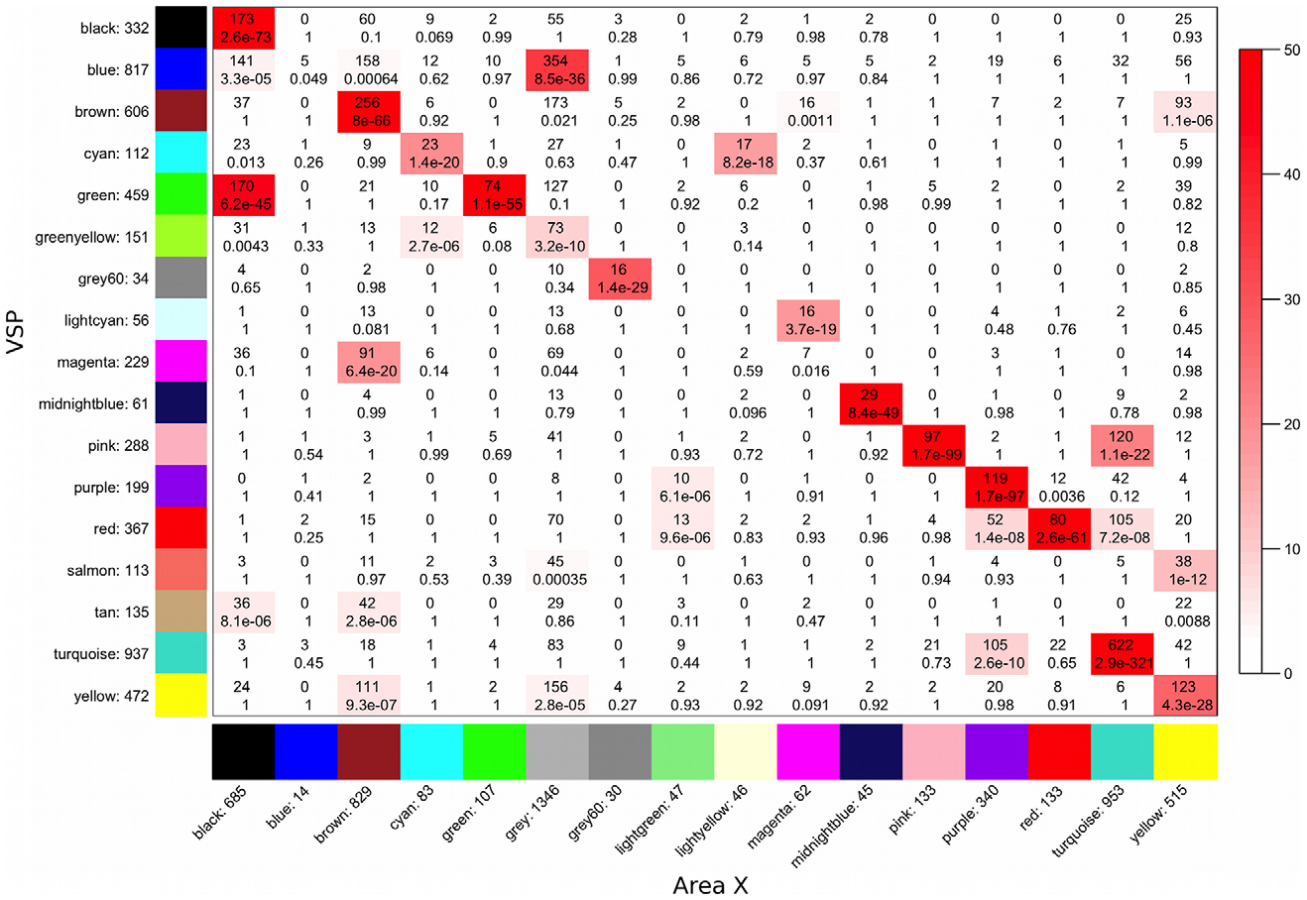
Overlap between VSP and area X co-expression modules. Cross-tabulation of VSP (rows, y-axis) and area X (columns, x-axis) module assignments. Each row and column is labeled by the corresponding module color and the total number of genes in the module. In the table, numbers show counts of genes in the intersection of the corresponding row and column module, i.e. shared between area X and VSP. The table is color coded by −log(p) of the Fisher's exact test p-value, according to the color legend on the right. The blue module is an example of a VSP module that is not well-preserved in area X, whereas the turquoise module is an example of a VSP module that is highly preserved in area X.

To enable direct comparisons between the VSP and area X networks and enhance reader-friendliness, we used the WGCNA function matchLabels() to re-assign the area X labels such that modules with significant overlap with a VSP module were assigned the same color label. The function overlapTable() was used to calculate overlap counts and Fisher's exact test p-values for the 2 sets of module assignments, and to produce **Figure 3**. The function modulePreservation() was used to compute module preservation statistics, and preservation rankings for VSP modules were determined based on these statistics, as described in [10].

### Module functional annotation

We screened modules for gene markers of neural cell-types and genes associated with PD and HD using lists obtained from [16] and the Ingenuity Knowledge Base (http://www.ingenuity.com/products/pathways_analysis.html), respectively. Enrichment p-values were computed via Fisher's exact test using the 11,482 genes remaining after pre-processing as the reference. The same 11,482 genes were also used as the reference for enrichment calculations in DAVID 6.7 (david.abcc.ncifcrf.gov) [17], which was used for module functional enrichment screening. Lists of genes from each module were converted to Entrez IDs to minimize ambiguity and uploaded to DAVID. Human Entrez IDs were used, as the zebra finch genome remains sparsely annotated, and multiple lines of evidence suggest that the neural systems supporting learned vocalization are highly analogous in humans and zebra finches [18]. For each module, terms with an enrichment p-value<0.1 were downloaded for further analysis.

Since some modules were enriched for hundreds of terms, many of which could be very similar to one another, we focused our investigations in the following ways: 1) To get a broad sense of the enrichment profile for each module, we used DAVID's functional annotation clustering tool to find groups of similar terms, based on genes shared between them, and ranked the clusters by the average enrichment level of terms they contained. 2) To prioritize individual enriched terms we first removed any with a false discovery rate (as computed by DAVID) greater than 15%, and removed those with fewer than 3 associated genes. Next, we defined a “term significance” (TS) score for each term as the average kME of genes annotated by the term, multiplied by 1 – p-value of the term, and ranked terms by these scores [9]. Our intent was to identify biological components and processes that were both highly enriched and represented by genes that most closely conformed to expression perturbations in the module.

The functional annotation results are meant to be treated as guides for further investigations and should be interpreted with caution since they represent comparisons of gene lists obtained from avian systems with mammalian databases. Annotation of the zebra finch genome is an ongoing process, making it important to return to our data in the future, including for analyses of probes that are currently unannotated or whose annotations have changed. Results in this paper were based on zebra finch microarray annotations from February 2011, see http://www.songbirdtranscriptome.net:8080/public.jsp for the most up-to-date annotations.

### Transcription factor analysis

The online Promoter Analysis and Interaction Network Tool (PAINT; www.dbi.tju.edu/dbi/tools/paint) [19] was used to identify transcription factor binding sequences (TFBSs) overrepresented in subsets of two VSP modules (blue, yellow). Human Entrez IDs for genes in these modules were uploaded to PAINT, where the upstream region (2,000 base pairs) of each gene was scanned for TFBSs using the MATCH algorithm and the public TRANSFAC database [20], [21]. The MATCH filter option “Minimize False Positives” was selected and the parameter “Core similarity threshold”, a measure of the quality of the match between a test sequence and the five most conserved positions in the TFBS position weight matrix, was set to 1. PAINT can identify TFBSs overrepresented in a subset of genes by comparison to a larger reference set and generate p-values using Fisher's exact test. We examined TFBSs overrepresented in subsets of the VSP blue and yellow modules based on their distribution in the area X network, using the entire module as the reference.

## Results

To determine whether gene co-regulation patterns underlie the functional specificities of sub-regions in the avian basal ganglia, we examined gene co-expression relationships in birds who sang varying amounts of song in the morning (0–1,270 motifs; motifs are stereotyped, neuroethologically relevant song sequences, [22]). RNA was extracted from area X and the VSP of each bird and hybridized to microarrays in order to generate the expression values used for constructing two gene networks; one from each sub-region. In addition to counting motifs, we computed the average values for each singing bird of 4 standard acoustic features used to characterize and compare birdsong recordings (mean pitch, pitch goodness, Wiener entropy, and frequency modulation) [23]. After array data pre-processing and outlier removal, *n* = 24 and *n* = 26 VSP and area X samples remained, respectively. Pre-processing and choice of genes for WGCNA were performed as previously described [9], with some additional filtering to optimize network construction and aid interpretation of the results (see Methods). Ultimately, 5,368 genes were retained in the final VSP network, and the corresponding probes for these genes were used to re-construct the area X network for comparison.

### Differences in VSP and area X network structure

WGCNA of the 5,368 genes retained after pre-processing revealed 17 VSP modules ranging in size from 34 to 937 genes (**Figure 2A**). Computing correlations between the MEs revealed a higher-order network of 3 meta-modules, visible as the 3 largest branches in the dendrogram in **Figure 2B–D**. Following network construction, we sought to identify any VSP-specific modules. Our previous area X-based study used statistical tests to quantify the likelihood of observing area X modules in the VSP, but we did not build an actual VSP network. Here, in addition to performing statistical tests of VSP module preservation, cross-tabulating module labels between the 2 networks allowed us to explore how functions enriched in VSP modules were split across the new area X network.

### ∼25% of genes in dense VSP network were background in area X

The re-constructed area X network consisted of 15 modules containing 4,022 of the 5,368 genes, ranging in size from 14 to 953 genes. The remaining 1,346/5,368 genes were not correlated strongly enough to genes in any of the 15 modules, and were thus considered background (denoted by the color grey in the area X module bar under the dendrogram in **Figure 2A**; also see **Table S1**). We note that genes referred to as “background” in the context of network construction are those with such weak correlations to other genes that they were not a part of any module. The VSP network was the result of iteratively culling grey background genes until none remained (see Methods), thus the fact that 1,346 of these genes were not a part of any module in area X is itself indicative of significant differences between area X and VSP transcription patterns during singing. In the previous study, when genes were selected for WGCNA based on their area X interconnectedness, only 193 of these 1,346 genes made it into that much larger original area X network (20,104 gene probes) [9]. In other words, these 1,346 genes were part of VSP co-expression groups that did not exist in area X, at least after 2 hours of singing. Next, since our primary interest was in studying co-expression patterns found in the VSP but not area X, we investigated which VSP modules these area X grey genes were members of to see if they were co-expressed in functionally significant groups.

### Background genes in area X were concentrated within a few modules in the VSP

Module colors in area X were assigned to match the VSP module assignments as closely as possible. This allowed us to compare the overlaps between VSP and area X module membership and identify the VSP module assignments of area X background (grey) genes (**Figure 3**). We observed significantly large subsets of most VSP modules in at least 1 area X module, e.g. the pink, purple, red, and turquoise modules. This implied a good deal of VSP module preservation in area X, however, some VSP modules were split across several modules in area X, suggesting they were composed of multiple interacting biological pathways that did not similarly interact in area X. As noted above, >1,300 genes were not in any proper area X module. The green-yellow, blue, salmon, and yellow VSP modules had the largest proportion of their genes fall into the group of grey background genes in area X (48%, 43%, 40%, and 33%, respectively). This comprised 60% of the area X grey genes, strongly suggesting that these modules were relatively specific to the VSP.

Next, we examined the distribution of VSP module genes across proper area X modules (i.e. disregarding area X grey genes), and for each VSP module, noted its highest proportion of genes in a single area X module. For example, 622 of 937 (66%) genes in the VSP turquoise module were also in the area X turquoise module, and 158 of 817 (19%) genes in the VSP blue module were in the area X brown module (**Figure 3**). In support of the idea that the green-yellow, blue, salmon, and yellow modules were relatively specific to the VSP, these 4 modules had lower average proportions of genes in a single proper area X module compared to the rest of the VSP modules (25% versus 42%, p = 0.017, Kruskal-Wallis). However, the assignment of a set of genes to the same module in both regions does not necessarily mean that the co-expression relationships among those genes are the same, or that any pathway they are a part of is functioning in the same way. Thus, to more closely investigate the preservation of VSP modules in area X, we computed module preservation statistics and derived a composite module preservation ranking for each module.

### VSP modules containing the most area X background genes were the most unique to VSP

Network statistics that describe co-expression patterns and connection strengths were computed within each VSP module and among the same genes in area X, regardless of their area X module assignment. Module preservation statistics were computed based on comparisons of these network statistics across the 2 regions and VSP modules were ranked based on the outcome [10]. In agreement with the module assignment comparisons, the yellow and blue modules were tied for least preserved, followed by the salmon and green-yellow modules. In contrast, the pink, purple, red, and turquoise modules were 4 of the 7 most preserved, and compared to the rest of the modules, were more preserved on average (p = 0.036, Kruskal-Wallis). Based on the correlation between their MEs, these 4 modules stood apart from the rest of the network in one large meta-module (**Figure 2B**). This is also apparent in the VSP network dendrogram (**Figure 2A**).

### Gene connectivity is a better indicator of tissue source than expression levels

**Figure 4:**
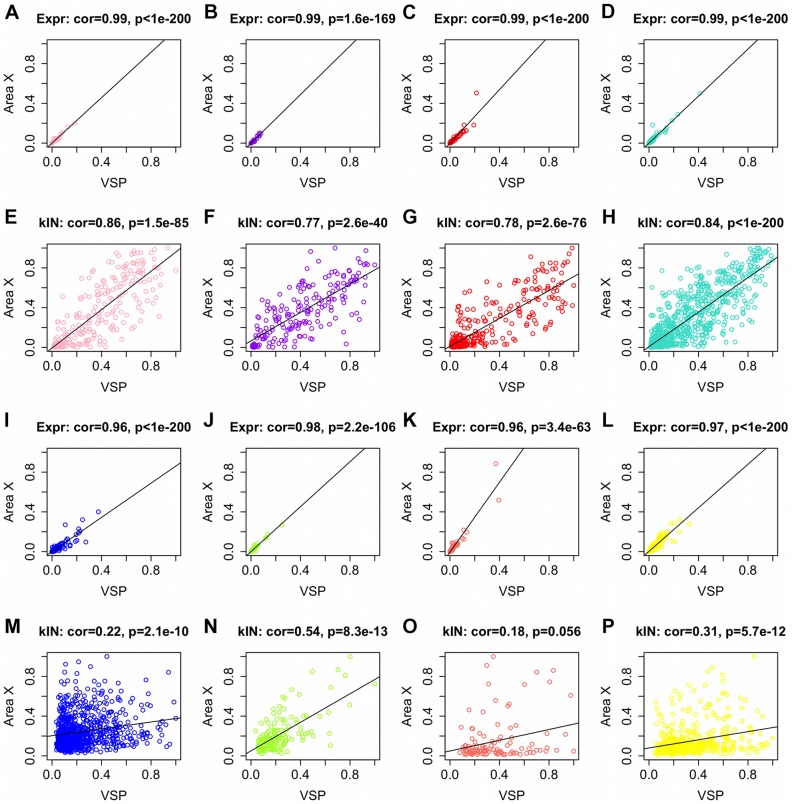
Preservation of VSP expression levels and intramodular connectivity in area X for selected modules. (**A–D**) Gene normalized median expression levels in area X are plotted as a function of levels in the VSP for the 4 modules most preserved in area X (left to right: pink, purple, red, turquoise), revealing extremely strong correlations, whereas intramodular connectivity values (kIN, **E–H**) were less correlated for the same genes. Spearman's rank correlation coefficient and corresponding p-value shown are shown above each plot. (**I–P**) Analogous plots of expression and kIN levels for genes in the 4 modules most specific to the VSP (left to right: blue, green-yellow, salmon, yellow), again revealing stronger correlations for expression levels than kIN, but note that kIN correlations are much stronger for the preserved (**E–H**) versus unpreserved (**M–P**) modules. kIN correlation was one of the preservation statistics used to rank VSP modules by their preservation in area X.

One statistic that figured in the preservation ranking calculations was the correlation between intramodular connectivity (kIN) in the VSP and area X. This statistic measures the preservation of intramodular hub gene status across the 2 areas [10]. Since the module preservation statistics were computed with reference to the VSP network, kIN in area X was computed using area X gene expression values, but based on VSP module assignments. Regardless of preservation ranking, normalized gene expression levels in the VSP and area X were nearly perfectly correlated (**Figure 4A–D, I–L**), whereas kIN values were more weakly correlated in the unpreserved versus preserved modules (**Figure 4E–H, M–P**). To more directly study the relationship between kIN and differential expression, standard t-tests were used to compare VSP and area X gene expression levels, and the results were compared to VSP and area X kIN (**Figure S2**). We found that high kIN is not a good predictor of differential expression, and perhaps counterintuitively, that the region in which a gene had more strong co-expression relationships was also the region in which it had relatively low expression levels. Thus, the differences between the VSP and area X cannot be accounted for by changes in expression levels per se, but are reflected well by co-expression network metrics (such as the correlation of kIN in the VSP and area X) that reflect coordinated changes in relative expression levels across groups of interacting genes.

### VSP modules showed mild but unexpected correlations to song features

**Figure 5:**
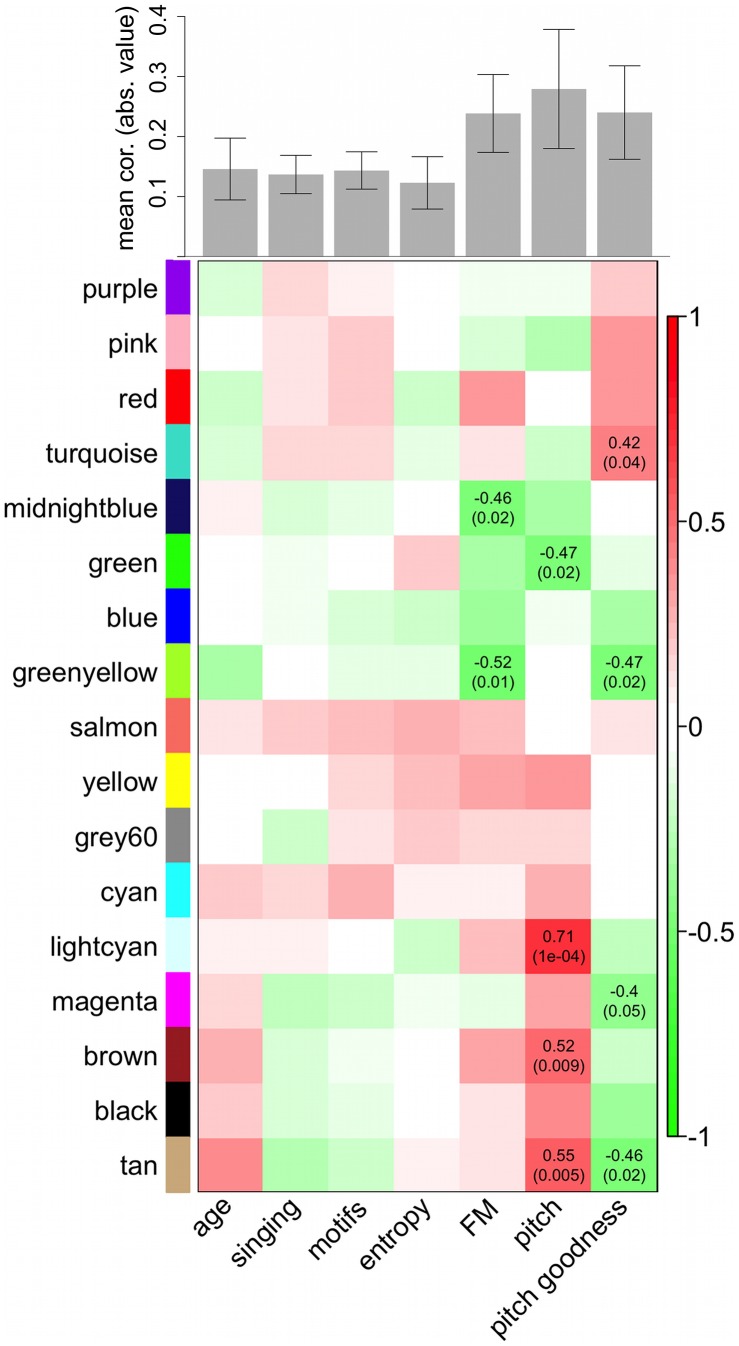
Relationships between VSP co-expression modules and quantitative traits. **Top:** Barplot showing average strength of VSP ME correlations to traits. Each bar represents the mean absolute value of correlations for the trait (column) in the heatmap it is above. Error bars denote 95% confidence intervals. Overall, correlation to traits important in area X (singing, motifs, entropy) was no stronger than to age, but there were surprisingly high correlations to FM, pitch, and pitch goodness. **Bottom:** Heatmap of correlations between VSP MEs (rows) and age and singing-related trait measurements (columns). Correlation coefficients with corresponding Student asymptotic p-values (parentheses) are shown in cells where p<0.05. Scale bar (right) indicates the range of possible correlations from positive (red, 1) to negative (green, −1).

To investigate the behavioral significance of the VSP modules, we computed correlations between MEs and 5 continuous singing measurements from each bird: number of motifs sung, pitch, pitch goodness (a measure of periodicity in the frequency spectrum), Wiener entropy (a measure of the width and uniformity of the spectrum), and frequency modulation (FM). We also tested one categorical grouping, whether the bird sang or not, and used the age of the bird at sacrifice as a non-behavioral phenotype for comparison to the singing traits. We did not expect to observe significant correlations between VSP MEs and singing measurements, a prediction that was largely validated; correlations were weak overall (**Figure 5**), especially compared to the strength of the correlations we observed in the large area X network in our previous study [9].

### VSP modules were most correlated to song features not important in area X

After computing the average absolute value of ME correlations to each trait we found that relationships to whether the bird sang or not, the number of motifs sung, and Wiener entropy were qualitatively no greater than to age (**Figure 5** barplot, top). This finding supports the functional specification seen in our previous area X study, where these 3 singing traits had highly significant correlations to MEs in 5 singing-related modules in our large area X network, and were predictive of module preservation in the VSP (stronger correlations = less preserved). Thus, we did not expect to find correlations between VSP modules and these song measures. We also re-computed ME correlations to singing and age for the new re-constructed area X modules and found that 3 modules were strongly correlated to the act and/or the amount of singing, replicating part of our previous findings (brown, midnight-blue, and yellow; these re-constructed area X modules were roughly analogous to the “song modules” in [9]; **Figure S3**).

In addition to these expected findings, we observed relatively strong average ME correlations to FM, pitch, and pitch goodness in the VSP, song features that were not strongly correlated to any MEs in either the previous or re-constructed area X network (**Figure 5**).

### Patterns of song correlations mapped to VSP meta-modules and module preservation in area X

**Figure 6:**
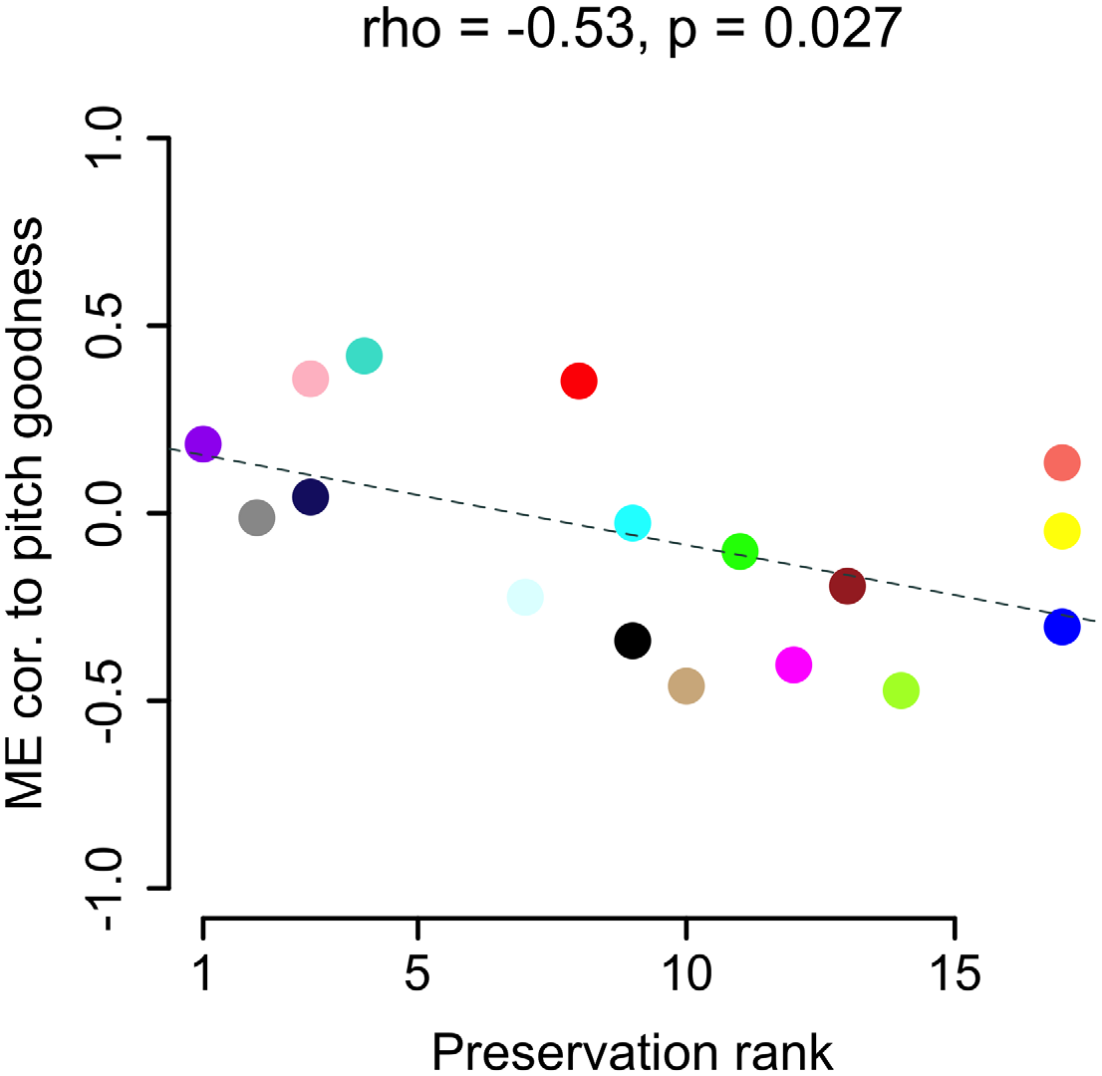
VSP module correlation to pitch goodness predicts preservation in area X. VSP ME correlations to pitch goodness (y-axis, same values as in pitch goodness column of heatmap in **Figure 5**) plotted as a function of module preservation rank in area X (x-axis, rank of 1 indicates most preserved). Each circle represents a module, colored accordingly, e.g. the blue module was one of the least preserved in area X and had a negative correlation to pitch goodness, while the turquoise module was one of the most preserved and had a positive correlation to pitch goodness. The dashed line represents the linear regression of ME-pitch goodness correlations on preservation rank, with Spearman's *rho* and p-value shown at top.

While the VSP ME correlations to FM, pitch, and pitch goodness were still weak compared to previous area X ME correlations to singing, number of motifs, and Wiener entropy, they were organized across the VSP modules in intriguing patterns (**Figure 5**). The blue, green, green-yellow, and midnight blue modules as a group showed more negative correlations to FM than the other modules (p = 0.0032, Kruskal-Wallis). Along with the fact that these made up the 2nd of the 3 VSP meta-modules (**Figure 2B**), this suggests that biological functions they represent may interact in some way related to, or reflective of, the amount of FM in a bird's song. Modules in meta-module 1 (pink, purple, red, and turquoise) had more positive correlations to pitch goodness as a group, while meta-modules 2 (blue, green, green-yellow, and midnight blue) and 3 (black, brown, cyan, grey60, light cyan, magenta, salmon, tan, and yellow) had more negative correlations (p = 0.013, Kruskal-Wallis), again implying functional groupings possibly related to a song spectral feature. The pitch goodness findings also bore a significant correlation to module preservation rankings; modules that were more preserved (e.g. those in meta-module 1) tended to show increased expression with increasing pitch goodness, i.e. positive correlations, while less preserved modules (e.g. blue and green-yellow) showed the opposite pattern (**Figure 6**). Finally, correlations to pitch were more positive for modules in meta-module 3 than in meta-modules 1 or 2 (p = 0.0024, Kruskal-Wallis).

### Biological significance and functional segregation in area X of VSP-specific modules

To identify biological functions and molecular pathways represented in the VSP modules, we used the functional annotation tools available through the Database for Annotation, Visualization, and Integrated Discovery (DAVID ver. 6.7) [17]. To focus our analysis of the results from DAVID, we filtered out enriched terms with false discovery rates (FDR)>15% and assigned the remaining terms in each module a “term significance” (TS) score, which effectively ranked terms by the influence their associated genes had in the module, scaled by the significance level of the term's enrichment (see Methods; also [9] for a version of TS that accounts for gene correlations to singing). We also screened modules for cell type markers and possible disease associations using Ingenuity and gene lists from the literature (see Methods; http://www.ingenuity.com/products/pathways_analysis.html; [16]).

Since the main goal of this study was to identify VSP-specific modules that could not have been found in our previous area X analysis, we focused on modules most specific to the VSP: the blue, green-yellow, salmon, and yellow modules. Based on the strength of their ME correlations, the blue and green-yellow modules were more similar to one another than to any other modules in the network (ME cor = 0.67, **Figure 2B**), and visual inspection of the network dendrogram suggests that the green-yellow module was a relatively distinct subset of the blue module (**Figure 2A**). A similar relationship existed between the salmon and yellow modules (ME cor = 0.74, **Figure 2B**), where salmon was a subset of yellow (**Figure 2A**). Genes in these 4 modules made up the majority of area X background (grey) genes (**Figure 3**), and based on preservation statistics, they were the least preserved in area X (**Figures 4** **and** **6**).

### VSP green-yellow module highly enriched for oligodendrocyte markers/myelination

**Figure 7:**
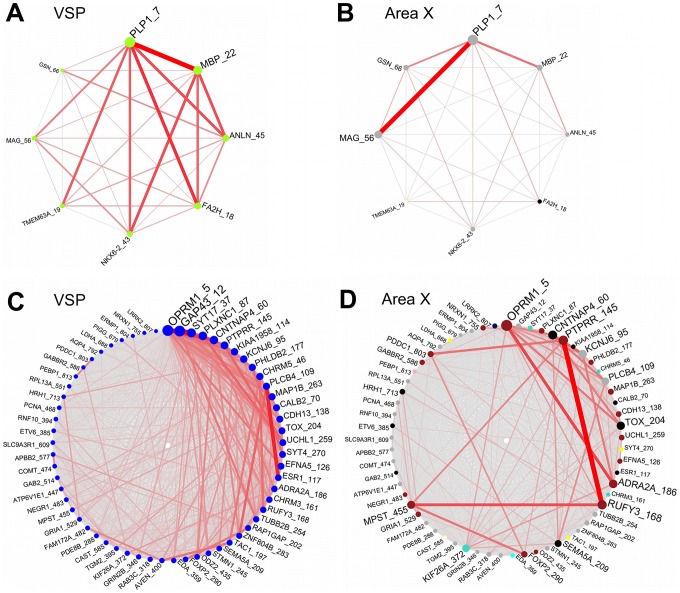
Schematics of connectivity among select VSP green-yellow and blue module genes in both regions. (**A**) Network connections are depicted between gene markers of oligodendrocytes in the VSP green-yellow module. Thicker/darker lines between nodes denote stronger connections. Node and label size represent overall connectivity among this group of genes (larger = more connected), nodes are colored by module assignment, and each gene symbol is appended (after underscore) by the rank of its kME value in the module, e.g. *PLP1* has the 7^th^ highest kME in the green-yellow module, *MBP* has the 22^nd^ highest kME, etc. Connections <0.1 are not shown. Area X connectivity between the same genes is shown in (**B**), where node and label size indicate connectivity among this group of genes in area X, and nodes are colored by area X module assignment. Visual inspection suggests that VSP connectivity patterns were not well-preserved in area X. Note that 6/8 genes were not in any area X module and were thus assigned the color “grey”. (**C–D**) Analogous plots depicting VSP and area X connections between genes associated with Parkinson's Disease in the VSP blue module: VSP (**C**), area X (**D**).

The green-yellow module was highly enriched for gene markers of oligodendrocytes (genes >10-fold enriched in oligos: p = 1.4e−6, Fisher's exact test) [16], e.g. proteolipid protein 1 (*PLP1*), fatty acid 2-hydroxylase (*FA2H*), and myelin basic protein (*MBP*). Commensurate with this finding, the 5 gene ontology (GO) terms in the green-yellow module with the highest TS scores were all related to myelination, including e.g., GO:0043209∼myelin sheath (**Table S2**). 6/8 oligodendrocyte markers from the green-yellow module were grey genes in area X, pointing to distinct co-expression relationships among these genes in the 2 brain regions (**Figure 7A–B**), suggesting that distinct myelination patterns may contribute to the different behavioral specifications of area X and the VSP in the songbird basal ganglia. Statistical tests of VSP module preservation in human caudate nucleus data [14] revealed that the green-yellow module was moderately preserved in human striatal tissue (Z_summary_ = 3.34, 2<Z_summary_<10 is considered moderate evidence of preservation [10]), suggesting that this module may represent myelination-related pathways important for motor behavior that are conserved within vertebrate basal ganglia.

### VSP blue module enriched for molecular functions disrupted in Parkinson's Disease

Dopaminergic input from the midbrain modulates the activity of medium spiny neurons in the avian basal ganglia, as in the mammalian basal ganglia [24], [25]. The death of midbrain dopaminergic cells in humans contributes to altered activity in these circuits resulting in vocal and non-vocal motor symptoms of Parkinson's Disease (PD) [26], [27]. The blue VSP module was highly enriched for genes involved in processes known to be disrupted in PD (p = 2.1e−5, Fisher's exact test), including many implicated in synaptic function and plasticity such as the mu opioid receptor (*OPRM1*) and growth associated protein 43 (*GAP43*). The connections among these blue module PD-associated genes were strikingly different in the area X network (**Figure 7C–D**), again highlighting distinct gene co-expression relationships in these subregions.

GO terms with the highest TS scores in the VSP blue module were related to G-protein signaling, specifically in relation to adenylate cyclase activity and cyclic AMP regulation, phospholipase C activity, and neurogenesis/axonogenesis (**Table S2**). Enrichments for most of the same functions were found among the PD-associated genes, with additional enrichments for terms related to behavior, learning, and memory (**Table S2**). Genes associated with these terms that had the highest blue module kME scores were *OPRM1*, speech-related *FOXP2*, 2 glutamate receptors (*GRIA1* and *GRIN2B*), tachykinin precursor 1 (*TAC1*), ubiquitin carboxyl-terminal esterase L1 (*UCHL1*), catechol-O-methyltransferase (*COMT*), and phosphatidylethanolamine binding protein 1 (*PEBP1*).

### VSP salmon and yellow modules enriched for molecular functions disrupted in Huntington's Disease

**Figure 8:**
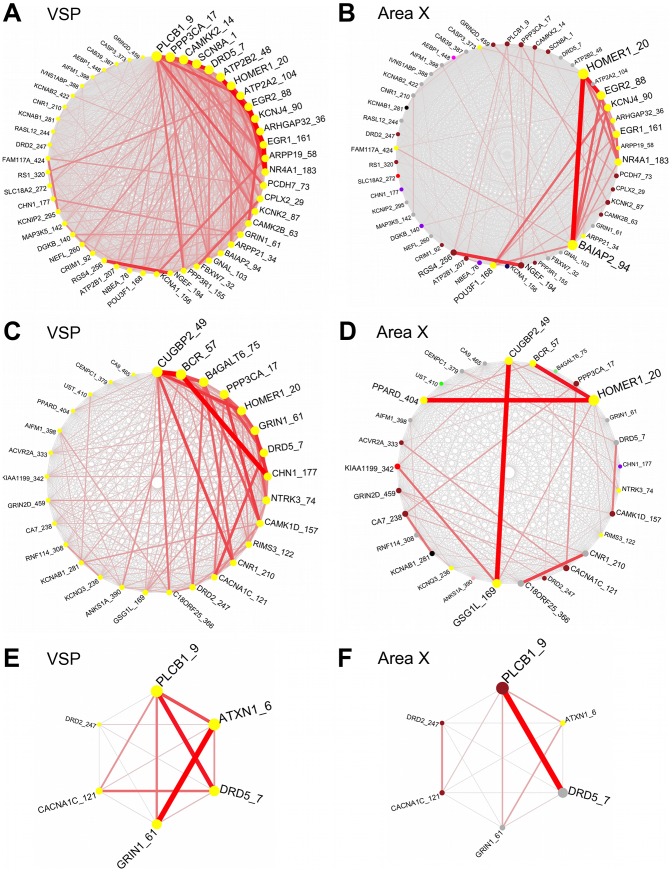
Schematics of connectivity among select VSP yellow module genes in both regions. Analogous plots to Figure 7, here showing VSP and area X connections between VSP yellow module genes associated with Huntington's disease: VSP (**A**), area X (**B**); Parkinson's disease: VSP (**C**), area X (**D**); and GO:0007612∼learning: VSP (**E**), area X (**F**).

Both the salmon and yellow VSP modules were enriched for genes associated with processes disrupted in Huntington's Disease (HD) which affects basal ganglia function through the death of medium spiny neurons (p = 0.006 and p = 2.6e−8, respectively, Fisher's exact test; **Figure 8A–B**) [28]. The same genes showed differential connection patterns in VSP compared to in area X. The yellow module also contained a high number of PD-associated genes (p = 0.049; **Figure 8C–D**). Terms with the highest TS scores in the salmon module were related to protein kinase, alternative splicing, and locomotory behavior (**Table S2**). The yellow module contained a significant number of genes that are members of known activity-driven pathways in the Kyoto Encyclopedia of Genes and Genomes database (KEGG) [29], including calcium signaling, MAPK signaling, and long-term potentiation (**Table S2**). In agreement with the enriched pathway findings, yellow module terms with the highest TS scores were almost exclusively related to voltage-gated potassium and calcium channel activity, potassium and calcium transport, and learning (**Figure 8E–F**). When we tested only the yellow module HD- or PD-associated genes, enrichments for the same functions were reiterated, with some interesting additional enriched terms such as GO:0008344∼adult locomotory behavior, GO:0050890∼cognition, GO:0007212∼dopamine receptor signaling pathway, and GO:0008306∼associative learning.

### VSP blue and yellow module genes split across area X modules in functionally significant groups

The VSP blue and yellow modules were the least preserved in area X. Most genes in the VSP blue module divided into 3 large groups in area X, with the largest number labeled as background (grey), and significant numbers in the brown and black area X modules (**Figure 3**). There were clear functional divisions between these groups: genes labeled grey in area X were enriched for catabolic processes and enzyme activation, genes in the area X brown module were enriched for cyclic AMP regulation and neurogenesis, and genes in the area X black module were enriched for steroid biosynthesis, response to steroid stimuli, the cell junction, and membrane-bound vesicles. These findings suggest that functional pathways working together in the VSP blue module, while still important in different area X modules, interact primarily with different sets of genes in area X. These differences may contribute to the contrasting types of motor behaviors supported by area X versus the VSP, for example singing versus nest-building [3].

Much like the blue module, the yellow module was split into 3 main groups in area X, with the largest number of genes labeled as background (grey), and significant numbers in the brown and yellow area X modules (**Figure 3**). After examining the enrichments in each of these portions of the VSP yellow module, clear functional distinctions again appeared: genes labeled grey in area X were enriched mostly for potassium channel activity/transport and ATPase activity, genes in the area X brown module were enriched for calcium channel activity/transport and gonadatropin-releasing hormone signaling, and genes in the area X yellow module showed enrichment for genes related to MAPK signaling. Interestingly, the area X yellow and brown modules, which contained significant numbers of VSP yellow and blue module genes, respectively, were highly correlated to singing and specific to area X (**Figure 3** **, Figure S3**). Since the yellow and blue VSP modules were the most specific to the VSP, this means that the modules that were most specific to each region none-the-less contained some of the same pathways. Presumably, distinct interactions of these pathways with other genes and pathways were what conferred module specificity in each brain region. In the discussion section, we assess the biological functions of key modules and then provide an analogy to illustrate how modular specificity could arise even when these modules share pathways.

We also examined transcription factor binding sequences (TFBSs) in the upstream sequences of VSP blue and yellow module genes (see Methods). Much like the functional distinctions we observed among subsets of these genes in different area X modules (and area X grey genes), we also found that specific TFBSs were overrepresented in each subset (**Figure S4**).

### Biological significance of well preserved modules

The pink, purple, red, and turquoise modules (meta-module 1; **Figure 2**) were relatively well preserved in area X (**Figures 4** **,** **6**). After performing the same FDR filtering as described for the unpreserved modules, and ranking terms by TS, the most prominent terms in these modules had to do with mitosis/cell division (purple), glycosylation (purple/turquoise), transcriptional regulation (purple), ion transport/binding (purple/pink/turquoise), the extracellular matrix (purple/turquoise), and development (red/turquoise). The turquoise module was enriched for known pathways associated with laminins, collagens and kinases (KEGG database; See **Table S3** for a full list of enriched terms in these modules.

## Discussion

In the songbird telencephalon, neurons that subserve learned vocal-motor communication are clustered together in what are referred to as “song control nuclei” within the larger brain structures of the cortex and basal ganglia. This arrangement permitted us to test the hypothesis that unique patterns of gene activation are associated with a given behavior. Accordingly, in a previous paper, we reported findings from area X where we identified gene modules driven by singing that were not present in the VSP [9]. Because the previous analysis centered on a relatively large area X network (20,104 gene probes), we did not construct an equivalent network in the VSP, and were thus unable to identify any VSP-specific modules, or examine how genes in area X-specific song modules were divided in the VSP. Here, we selected genes for WGCNA based on their interconnectedness in the VSP; comparatively stringent gene filtering criteria resulted in a much smaller network than in the previous analysis (5,368 versus 20,104 gene probes), which made it feasible to re-construct an area X network and make direct comparisons of module assignments. This allowed us to compare functional enrichment results in subsets of VSP modules that were split across multiple modules in area X, providing an initial description of how molecular pathways interact differently in the 2 anatomically adjacent, but functionally distinct sub-regions.

We did not expect VSP co-expression patterns to relate to singing, an expectation that was met for song measures that were strongly correlated to modules in area X (e.g. number of motifs sung). Surprisingly though, we found that module correlations to pitch goodness were predictive of preservation in area X; less preserved modules tended to contain genes whose expression decreased with increasing pitch goodness, while the opposite was true for more preserved modules. There were also patterns of weak correlations to FM, particularly for modules in meta-module 2, which included the VSP-specific blue and green-yellow modules. These findings suggest that molecular processes supported by the VSP-specific modules have some bearing on organization of the song frequency spectrum, even though unlike area X, the VSP is not thought to be specialized for vocal-motor learning and production. Alternatively, VSP-singing correlations could reflect changes in body posture or other movements [5] that might affect the quality and modulation of the frequency spectrum, or simply the proximity of the VSP to area X; related molecular activity throughout the surrounding striato-pallidum could be a side-effect of the powerful singing-driven changes seen in area X. The latter seems unlikely however, since the VSP modules were most correlated to the exact song features that did not show significant correlations in area X. We previously predicted that application of WGCNA to expression data from additional song nuclei, e.g. those in cortical areas, would reveal song-regulated gene ensembles not found in neighboring tissue [9]. Relevant to our present findings, studying the relationships between singing and modules specific to tissue neighboring other song regions, e.g. HVC and the outlying nidopallium, could help make sense of the weak module-singing correlations we observed here in the VSP.

Cross-subregion comparison of module assignments and statistical tests of VSP module preservation highlighted the blue, green-yellow, salmon, and yellow modules as the most specific to the VSP. The green-yellow module was highly enriched for genes involved in myelination that were not similarly co-expressed in area X, suggesting that distinct myelination patterns may contribute to the functional differences between area X and the VSP, perhaps by shaping the efficiency or temporal precision of axonal communication in neuronal networks that more directly affect behavior.

In the VSP, many of the blue module's most significantly enriched functions were focused around G-protein coupled receptor mediated signaling, specifically in relation to intracellular calcium (adenylate cyclase activity, cyclic AMP regulation, phospholipase C activity). In the yellow module, voltage-gated calcium and potassium ion channel activity and MAPK signaling were its most significantly enriched functions. Many genes associated with learning and behavior in these modules code for dopamine, opioid, and glutamate receptors (AMPA and NMDA-types). These enrichments make sense in light of what is known about molecular function in the mammalian striatum, where glutamate receptor activation activates phospholipase C, initiating a host of cellular processes, some of which have a regulatory effect on dopamine signaling. Dopamine in turn modulates glutamatergic inputs to the cell, and coordinated dopamine and glutamate signaling enable corticostriatal long term potentiation (LTP). MAPK signaling is also sensitive to glutamate receptor activity, and MAPK activation may regulate striatal activity and goal-directed behavior through phosphorylation of inwardly rectifying potassium channels [1].

The blue and yellow modules were both enriched for genes associated with processes disrupted in PD and HD, including functions related to synaptic transmission/plasticity, learning, and behavior. Among all of the PD-associated genes in these modules, the gene coding for the mu opioid receptor (*OPRM1*) was the most highly interconnected, an interesting finding given that opioid receptor signaling is known to affect adenylate cyclase, voltage-gated calcium channels, potassium conductance, transmitter release, MAPK, and protein kinase C [30]. Although cellular pathology and changes in neural activity of the medium spiny neurons have been investigated in genetic models of PD and HD, molecular interactions underlying abnormal motor behavior have not been as well studied, thus our characterization of co-regulation among PD and HD associated genes can provide a baseline for comparison in future work. Furthermore, future studies can explore the significance of the differential patterns of interactions among these genes in area X versus VSP (**Figures 7** **,** **8**), which may convey selective activation of molecular pathways for vocal versus non-vocal motor behavior. Given that these diseases have both vocal and non-vocal motor components, comparison of these modules to those in striatal gene co-expression networks in pathological tissue would help determine which functions and pathway interactions are most disrupted during the disease, including providing insight into interactions between dopaminergic and non-dopaminergic mechanisms. In addition, since there were so many blue and yellow module PD/HD-associated genes contributing to the above biological processes, other genes in the same pathways, or strongly connected to them, could be considered “guilty by association”, and may also play a role in these pathologies [14].

More generally, our findings supported our predictions and speak to the emerging view that massive transcriptional changes can induce shifts between distinct “neurogenomic states” that underlie specific behaviors [31]. Thousands of genes are co-expressed in distinct patterns in area X and the VSP. Specific patterns in area X are highly correlated to the region's specialty, singing, while VSP-specific patterns contain many genes implicated in PD/HD and are involved in processes that are disrupted in these pathologies. For example, gene groups from the VSP yellow and blue modules involved in calcium channel activity (yellow) and cyclic AMP regulation (blue) were found in the re-constructed area X brown module, which was highly correlated to singing. This means that genes involved in these functions were important in modules that were most specific to their respective striato-pallidal sub-regions (yellow/blue in VSP, brown in area X) and had very different relationships to singing. One interpretation of these findings is that functions like calcium channel activity and cyclic AMP regulation are co-regulated with distinct sets of other functions in the VSP versus area X, thus contributing to the behavioral specializations of these areas, an idea supported by the fact that we observed clear functional segregation of VSP yellow and blue module genes across other area X modules. For example, VSP blue module genes related to membrane-bound vesicles were found in the re-constructed area X black module, whereas VSP blue module genes involved in cyclic AMP regulation were found in the area X brown singing-related module. This may imply that within the VSP blue module, the interaction between genes involved in membrane-bound vesicles and those for cyclic AMP regulation is critical for PD-related non-vocal motor function. In contrast, in area X, the cyclic AMP regulation may be less related to membrane bound vesicles but important for vocal-motor function.

The following analogy may help make the preceding point more clear. Think of the different biological functions as musicians, the striato-pallidal subregions as performance venues, and the different types of motor behaviors supported by these regions as styles of music. For example, a guitar player, bassist, drummer, and vocalist performing together in one venue play hard rock, but the same drummer and bass player performing alongside a pianist and saxophone player at the other venue might play bebop jazz. The presence of, e.g., the drummer alone does not determine the style of music being played, nor can a venue specialize in a particular style, e.g. a jazz club, without the right combination of players performing together. Stylistically appropriate types of interaction between particular musicians are what determines the musical form. Thus, for example, the enrichment for cyclic AMP regulation in the VSP blue module does not mean that module is necessarily important for vocal-motor behavior, even though cyclic AMP regulation is also enriched in the area X brown module, which was highly singing-related. It depends on when and where cyclic AMP activity is being regulated, and with what other functions it is regulated in concert with.

Together, these results suggest that the expression of a single gene or the activation/inhibition of a single pathway is not sufficient to underlie the functional specificities in area X and the VSP. Better comprehension of the molecular complexity underlying behavior in the basal ganglia will require an understanding of the dynamic interactions between many genes and pathways. WGCNA-type approaches provide snapshots of these interactions at a given time, and can help generate hypotheses about network function and the relative importance of single genes/pathways.

## Supporting Information

Figure S1 **VSP module robustness confirmed by quality scores.** Horizontal bars correspond to module quality Z_summary_ scores (x-axis) for VSP gene co-expression modules, as computed by the WGCNA library modulePreservation() function (Langfelder et al., 2008). The dark red vertical line at Z_summary_ = 10 corresponds to the threshold for high module quality. High quality modules contain densely interconnected genes and are well-separated from other modules in the network. All VSP modules have scores >10, indicating a high degree of module robustness and reproducibility throughout the VSP (see WGCNA section of Methods and Langfelder et al., 2011).
(TIFF)

Figure S2 **Relationships between differential expression and intramodular connectivity.** The standard t-test statistic (y-axis) is plotted as a function of kIN in the VSP (A; kIN.VSP, x-axis) and the difference between VSP and area X kIN (B; kIN.VSP – kIN.X, x-axis). Positive values of the t-test statistic indicate relative over-expression in the VSP, and vice versa. Each circle represents a single gene, colored by VSP module assignment, and the Pearson correlation coefficient with p-value (based on Fisher's *z* transformation) is reported above each plot. (**A**) Vertical lines at 0.48 and 0.77 denote the 80^th^ and 95^th^ percentiles for kIN.VSP, and horizontal lines at −5 and 5 denote t-test critical values at p<0.00001, which approximates the Bonferroni corrected significance threshold for the t-tests (0.05/5,368 genes ∼ = 0.000009). Overall, there is no correlation between kIN.VSP and the t-test statistic used for differential gene expression, and while some high kIN genes exhibit significant t-test outcomes, most do not. Also, a large number of low kIN genes are highly differentially expressed. (**B**) Vertical lines at −0.34 and 0.49 indicate boundaries for the 95^th^ percentile of genes with the largest differences in kIN. and horizontal lines at −5 and 5 again denote t-test critical values at p<0.00001. There was a significant, but very weak, relationship between t-test outcome and differential connectivity, such that genes with higher kIN in the VSP tended to have higher expression levels in area X compared to VSP. Many genes with strong evidence for differential expression are not differentially connected, and vice versa.
(TIFF)

Figure S3 **Relationships between area X co-expression modules and quantitative traits.** Heatmap of correlations between re-constructed area X MEs (rows) and age and singing-related trait measurements (columns). Numbers report the correlation coefficients and Student asymptotic p-value (parentheses) for relationships where p<0.05. Scale bar (right) indicates the range of possible correlations from positive (red, 1) to negative (green, −1). The brown, midnight-blue, and yellow modules had significant correlations to the act and/or the amount of singing, and were roughly analogous to the “song modules” in Hilliard et al., 2012 (brown and midnight-blue ∼ = dark green/orange song modules, yellow ∼ = blue song module).
(TIFF)

Figure S4 **Enrichment of specific transcription factor binding sequences (TFBSs) in functionally distinct subsets of VSP-specific modules.** Heatmaps of enrichment p-values for TFBSs (rows) in subsets of the genes from the VSP-specific blue (A) and yellow (B) modules, segregated by their area X module assignments (columns). Previous analysis of results from DAVID found that, based on their distribution in area X modules, subsets of the VSP blue and yellow modules were enriched for distinct sets of biological functions. Actual p-values are shown in cells with p<0.05, as determined via Fisher's exact test by comparison to TFBSs over-represented in the module as a whole. An important caveat is that these results were based on TFBSs and upstream sequences from the human genome, since PAINT (Promoter Analysis and Interaction Network Tool, www.dbi.tju.edu/dbi/tools/paint; Vadigepalli et al., 2003) is currently limited to human, mouse, and rat data. Multiple lines of evidence suggest that the neural systems supporting learned vocalization are highly analogous in humans and zebra finches (Jarvis, 2004), thus these sequences may or may not be the same in birds. In light of this, the enrichment for the FOXJ2_02 transcription factor in the area X brown module, which is highly singing-related (see Figure S3), is potentially interesting since its core binding sequence is very similar to that of FOXP2 (Pérez-Sánchez et al., 2000; Spiteri et al., 2007), a well known singing-related transcription factor.
(TIFF)

Table S1 **Network summary data.** Information pertaining to all genes selected for VSP network construction, with the columns organized as follows. **Columns A–E:** Probe (col. A) and clone (col. B) ID numbers, probe nucleotide sequence (col. C), gene symbol (col. D) and name (col. E), Gene symbols and names reflect microarray annotations as of February 2011, see http://www.songbirdtranscriptome.net:8080/public.jsp for the most up-to-date annotations. **Columns F–G:** Module assignments in the VSP reference network (col. F) and the re-constructed area X network (col. G). **Columns H–AI:** Gene significance (GS) information for age and 6 song traits, as measured in the VSP and area X. Correlations are shown along with their associated FDR-corrected p-values, i.e. q-values. Each trait is represented by a block of 4 columns; the first 2 depict GS and q-values as measured in the VSP (col. names end with .VSP), the next 2 depict GS and q-values as measured in area X (col. names end with .X). GS measurements with q<0.05 were considered significant. **Columns AJ–AZ:** Module eigengene-based connectivity (kME) for each gene in every VSP module. **Columns BA–BP:** kME for each gene in every area X module.
(XLS)

Table S2 **Functional annotation results for VSP-specific modules.** Functional annotation results for the VSP modules least preserved in area X (blue, green-yellow, salmon, and yellow). Each row represents an enriched term, columns represent the module the term was enriched in (Module), the database/category the term is from (Category), the term itself (Term), whether the term was enriched only in the module indicated in the first column (uniqueToMod), the number of module genes annotated by the term (Count), the enrichment p-value (PValue) and false discovery rate (FDR) computed by DAVID, the average module membership of genes annotated by the term (avg.kME), the term significance score as computed by the average kME multiplied by 1 – the enrichment p-value (kME*(1-pval)), and the genes annotated by the term (geneSym).
(XLS)

Table S3 **Functional annotation results for modules common to VSP and area X.** Same as Table S1, but for the VSP modules most preserved in area X (pink, purple, red, and turquoise).
(XLS)

Text S1 **References for supporting information.**
(DOC)

## References

[1] Shiflett MW, Balleine BW (2011) Molecular substrates of action control in cortico-striatal circuits. Prog Neurobiol 95: 1–13. 21704115 10.1016/j.pneurobio.2011.05.007 PMC3175490

[2] Reiner A, Perkel DJ, Bruce LL, Butler AB, Csillag A, et al (2004) Revised nomenclature for avian telencephalon and some related brainstem nuclei. J Comp Neurol 473: 377–414. 15116397 10.1002/cne.20118 PMC2518311

[3] Walters MJ, Harding CF (1988) The effects of an aromatization inhibiter on the reproductive behavior of male zebra finches. Horm Behav 22: 207–18. 3397053 10.1016/0018-506x(88)90067-0

[4] Williams H (2001) Choreography of song, dance and beak movements in the zebra finch (Taeniopygia guttata). J Exp Biol 204: 3497–506. 11707499 10.1242/jeb.204.20.3497

[5] Feenders G, Liedvogel M, Rivas M, Zapka M, Horita H, et al (2008) Molecular mapping of movement-associated areas in the avian brain: A motor theory for vocal learning origin. PloS One 3: e1768. 18335043 10.1371/journal.pone.0001768 PMC2258151

[6] Zhang B, Horvath S (2005) A general framework for weighted gene co-expression network analysis. Statistical Applications in Genetics and Molecular Biology 4: Article17. 16646834 10.2202/1544-6115.1128

[7] Miller JA, Horvath S, Geschwind DH (2010) Divergence of human and mouse brain transcriptome highlights Alzheimer disease pathways. Proc Natl Acad Sci U S A 107: 12698–703. 20616000 10.1073/pnas.0914257107 PMC2906579

[8] Oldham MC, Horvath S, Geschwind DH (2006) Conservation and evolution of gene co-expression networks in human and chimpanzee brain. Proc Natl Acad Sci USA 103: 17973–8. 17101986 10.1073/pnas.0605938103 PMC1693857

[9] Hilliard AT, Miller JE, Fraley E, Horvath S, White SA (2012) Molecular microcircuitry underlies functional specification within a basal ganglia circuit dedicated to vocal learning. Neuron 73: 537–52. 22325205 10.1016/j.neuron.2012.01.005 PMC3278710

[10] Langfelder P, Luo R, Oldham MC, Horvath S (2011) Is my network module preserved and reproducible? PloS Comp Biol 7: e1001057. 10.1371/journal.pcbi.1001057 PMC3024255 21283776

[11] Langfelder P, Horvath S (2008) WGCNA: an R package for weighted correlation network analysis. BMC Bioinformatics 9: 559. 19114008 10.1186/1471-2105-9-559 PMC2631488

[12] Miller JA, Cai C, Langfelder P, Geschwind DH, Kurian SM, et al (2011) Strategies for aggregating gene expression data: The collapseRows R function. BMC Bioinformatics 12: 322. 21816037 10.1186/1471-2105-12-322 PMC3166942

[13] Yip AM, Horvath S (2007) Gene network interconnectedness and the generalized topological overlap measure. BMC Bioinformatics 8: 22. 17250769 10.1186/1471-2105-8-22 PMC1797055

[14] Oldham MC, Konopka G, Iwamoto K, Langfelder P, Kato T, et al (2008) Functional organization of the transcriptome in human brain. Nat Neurosci 11: 1271–1282. 18849986 10.1038/nn.2207 PMC2756411

[15] Langfelder P, Zhang B, Horvath S (2007) Defining clusters from a hierarchical cluster tree: the Dynamic Tree Cut package for R. Bioinformatics 24: 719–720. 18024473 10.1093/bioinformatics/btm563

[16] Cahoy JD, Emery B, Kaushal A, Foo LC, Zamanian JL, et al (2008) A transcriptome database for astrocytes, neurons, and oligodendrocytes: a new resource for understanding brain development and function. J Neurosci 28: 264–278. 18171944 10.1523/JNEUROSCI.4178-07.2008 PMC6671143

[17] Huang W, Sherman BT, Lempicki RA (2009) Systematic and integra- tive analysis of large gene lists using DAVID bioinformatics resources. Nat Protoc 4: 44–57. 19131956 10.1038/nprot.2008.211

[18] Jarvis ED (2004) Learned birdsong and the neurobiology of human language. Ann NY Acad Sci 1016: 749–777. 15313804 10.1196/annals.1298.038 PMC2485240

[19] Vadigepalli R, Chakravarthula P, Zak DE, Schwaber JS, Gonye GE (2003) PAINT: a promoter analysis and interaction network generation tool for gene regulatory network identification. OMICS 7: 235–52. 14583114 10.1089/153623103322452378

[20] Kel AE, Gössling E, Reuter I, Cheremushkin E, Kel-Margoulis OV, et al (2003) MATCH: A tool for searching transcription factor binding sites in DNA sequences. Nucleic Acids Res 31: 3576–9. 12824369 10.1093/nar/gkg585 PMC169193

[21] Matys V, Fricke E, Geffers R, Gössling E, Haubrock M, et al (2003) TRANSFAC: transcriptional regulation, from patterns to profiles. Nucleic Acids Res 31: 374–8. 12520026 10.1093/nar/gkg108 PMC165555

[22] Hahnloser RHR, Kozhevnikov AA, Fee MS (2002) An ultra-sparse code underlies the generation of neural sequences in a songbird. Nature 419: 65–70. 12214232 10.1038/nature00974

[23] Tchernichovski O, Nottebohm F, Ho CE, Pesaran B, Mitra PP (2000) A procedure for an automated measurement of song similarity. Anim Behav 59: 1167–1176. 10877896 10.1006/anbe.1999.1416

[24] Soha JA, Shimizu T, Doupe AJ (1996) Development of the catecholaminergic innervation of the song system of the male zebra finch. J Neurobiol 29: 473–89. 8656212 10.1002/(SICI)1097-4695(199604)29:4<473::AID-NEU5>3.0.CO;2-5

[25] Ding L, Perkel DJ (2002) Dopamine modulates excitability of spiny neurons in the avian basal ganglia. J Neurosci 22: 5210–8. 12077216 10.1523/JNEUROSCI.22-12-05210.2002 PMC6757730

[26] Sapir S, Ramig L, Fox C (2008) Speech and swallowing disorders in Parkinson disease. Curr. Opin. Otolaryngol. Head Neck Surg 16: 205–10. 10.1097/MOO.0b013e3282febd3a 18475072

[27] Wichmann T, Dostrovsky JO (2011) Pathological basal ganglia activity in movement disorders. Neuroscience 198: 232–44. 21723919 10.1016/j.neuroscience.2011.06.048 PMC3209553

[28] Raymond LA, André VM, Cepeda C, Gladding CM, Milnerwood AJ, et al (2011) Pathophysiology of Huntington's disease: time-dependent alterations in synaptic and receptor function. Neuroscience 198: 252–73. 21907762 10.1016/j.neuroscience.2011.08.052 PMC3221774

[29] Kanehisa M, Goto S (2000) KEGG: kyoto encyclopedia of genes and genomes. Nucleic Acids Res 28: 27–30. 10592173 10.1093/nar/28.1.27 PMC102409

[30] Samadi P, Bédard PJ, Rouillard C (2006) Opioids and motor complications in Parkinson's disease. Trends Pharmacol Sci 27: 512–7. 16908075 10.1016/j.tips.2006.08.002

[31] Chandrasekaran S, Ament SA, Eddy JA, Rodriguez-Zas SL, Schatz BR, et al (2011) Behavior-specific changes in transcriptional modules lead to distinct and predictable neurogenomic states. Proc Natl Acad Sci U S A 108: 18020–5. 21960440 10.1073/pnas.1114093108 PMC3207651

[32] Roberts TF, Klein ME, Kubke MF, Wild JM, Mooney R (2008) Telencephalic neurons monosynaptically link brainstem and forebrain premotor networks necessary for song. J Neurosci 28: 3479–89. 18367614 10.1523/JNEUROSCI.0177-08.2008 PMC2843410

