# Supplementary figures and images for: Distinct Neurogenomic States in Basal Ganglia Subregions Relate Differently to Singing Behavior in Songbirds
Source: PLoS Comput Biol. 2012 Nov 8;8(11):e1002773. doi: 10.1371/journal.pcbi.1002773 (PMC3493463; doi:10.1371/journal.pcbi.1002773)

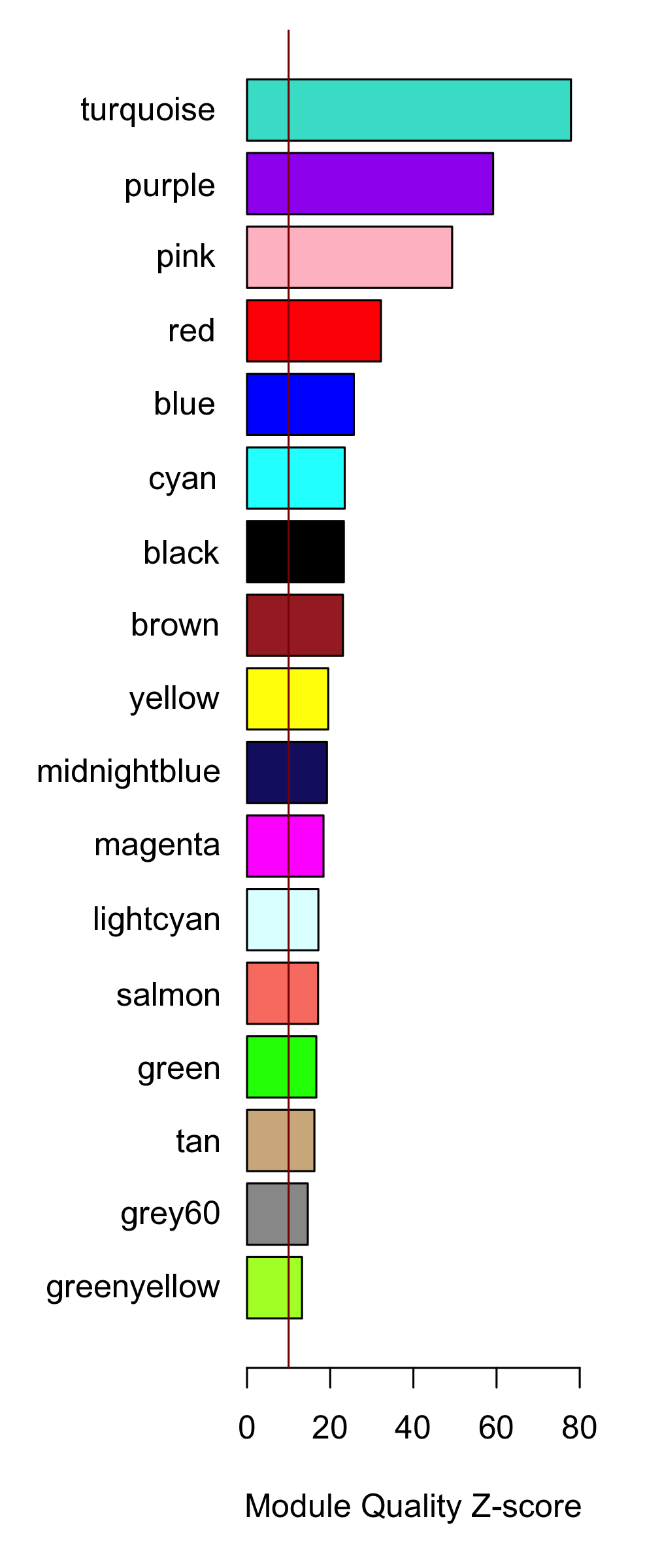

Supplement: Figure S1 — VSP module robustness confirmed by quality scores. Horizontal bars correspond to module quality Zsummary scores (x-axis) for VSP gene co-expression modules, as computed by the WGCNA library modulePreservation() function (Langfelder et al., 2008). The dark red vertical line at Zsummary = 10 corresponds to the threshold for high module quality. High quality modules contain densely interconnected genes and are well-separated from other modules in the network. All VSP modules have scores >10, indicating a high degree of module robustness and reproducibility throughout the VSP (see WGCNA section of Methods and Langfelder et al., 2011). (TIFF) [file pcbi.1002773.s001.tif]

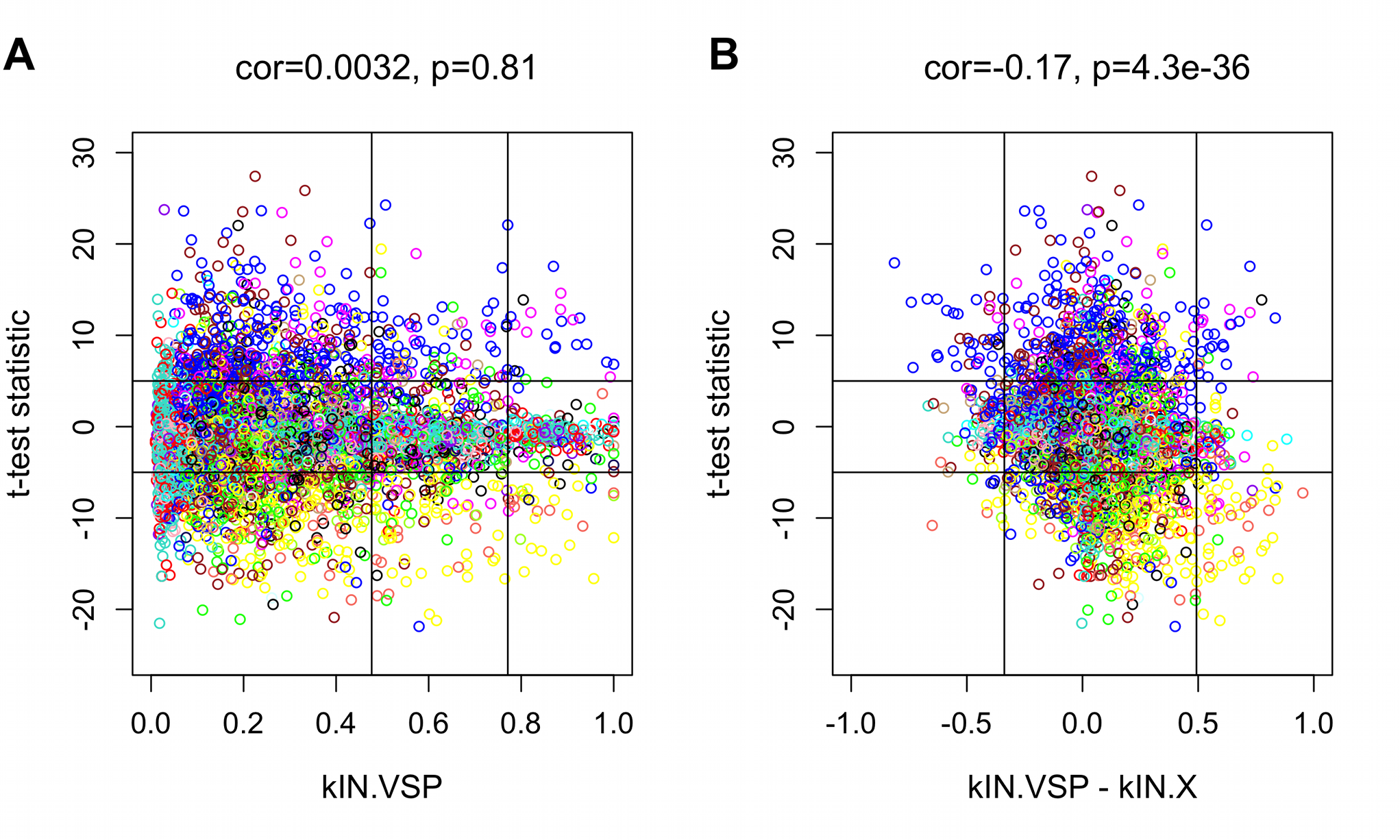

Supplement: Figure S2 — Relationships between differential expression and intramodular connectivity. The standard t-test statistic (y-axis) is plotted as a function of kIN in the VSP (A; kIN.VSP, x-axis) and the difference between VSP and area X kIN (B; kIN.VSP – kIN.X, x-axis). Positive values of the t-test statistic indicate relative over-expression in the VSP, and vice versa. Each circle represents a single gene, colored by VSP module assignment, and the Pearson correlation coefficient with p-value (based on Fisher's z transformation) is reported above each plot. (A) Vertical lines at 0.48 and 0.77 denote the 80th and 95th percentiles for kIN.VSP, and horizontal lines at −5 and 5 denote t-test critical values at p<0.00001, which approximates the Bonferroni corrected significance threshold for the t-tests (0.05/5,368 genes ∼ = 0.000009). Overall, there is no correlation between kIN.VSP and the t-test statistic used for differential gene expression, and while some high kIN genes exhibit significant t-test outcomes, most do not. Also, a large number of low kIN genes are highly differentially expressed. (B) Vertical lines at −0.34 and 0.49 indicate boundaries for the 95th percentile of genes with the largest differences in kIN. and horizontal lines at −5 and 5 again denote t-test critical values at p<0.00001. There was a significant, but very weak, relationship between t-test outcome and differential connectivity, such that genes with higher kIN in the VSP tended to have higher expression levels in area X compared to VSP. Many genes with strong evidence for differential expression are not differentially connected, and vice versa. (TIFF) [file pcbi.1002773.s002.tif]

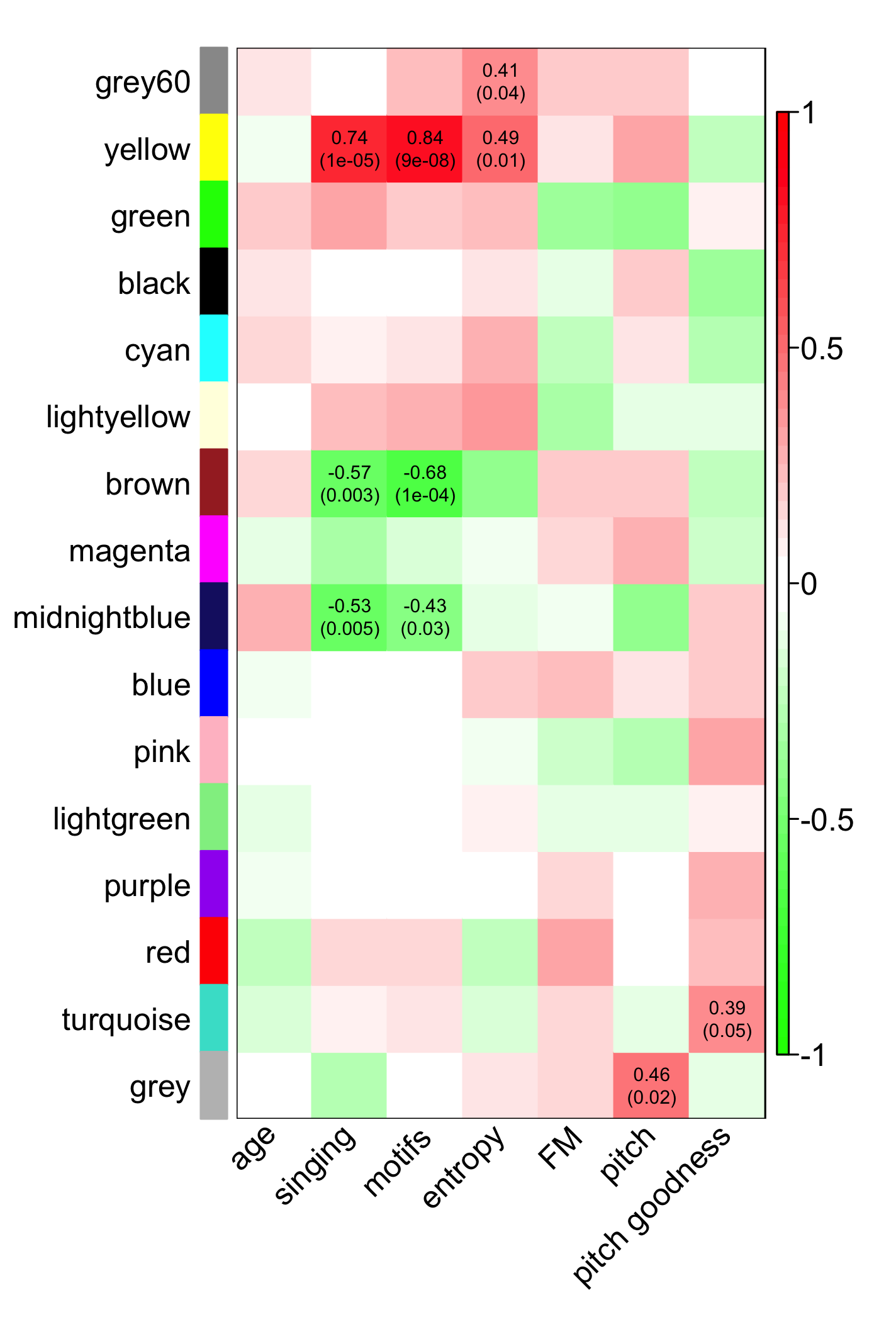

Supplement: Figure S3 — Relationships between area X co-expression modules and quantitative traits. Heatmap of correlations between re-constructed area X MEs (rows) and age and singing-related trait measurements (columns). Numbers report the correlation coefficients and Student asymptotic p-value (parentheses) for relationships where p<0.05. Scale bar (right) indicates the range of possible correlations from positive (red, 1) to negative (green, −1). The brown, midnight-blue, and yellow modules had significant correlations to the act and/or the amount of singing, and were roughly analogous to the “song modules” in Hilliard et al., 2012 (brown and midnight-blue ∼ = dark green/orange song modules, yellow ∼ = blue song module). (TIFF) [file pcbi.1002773.s003.tif]

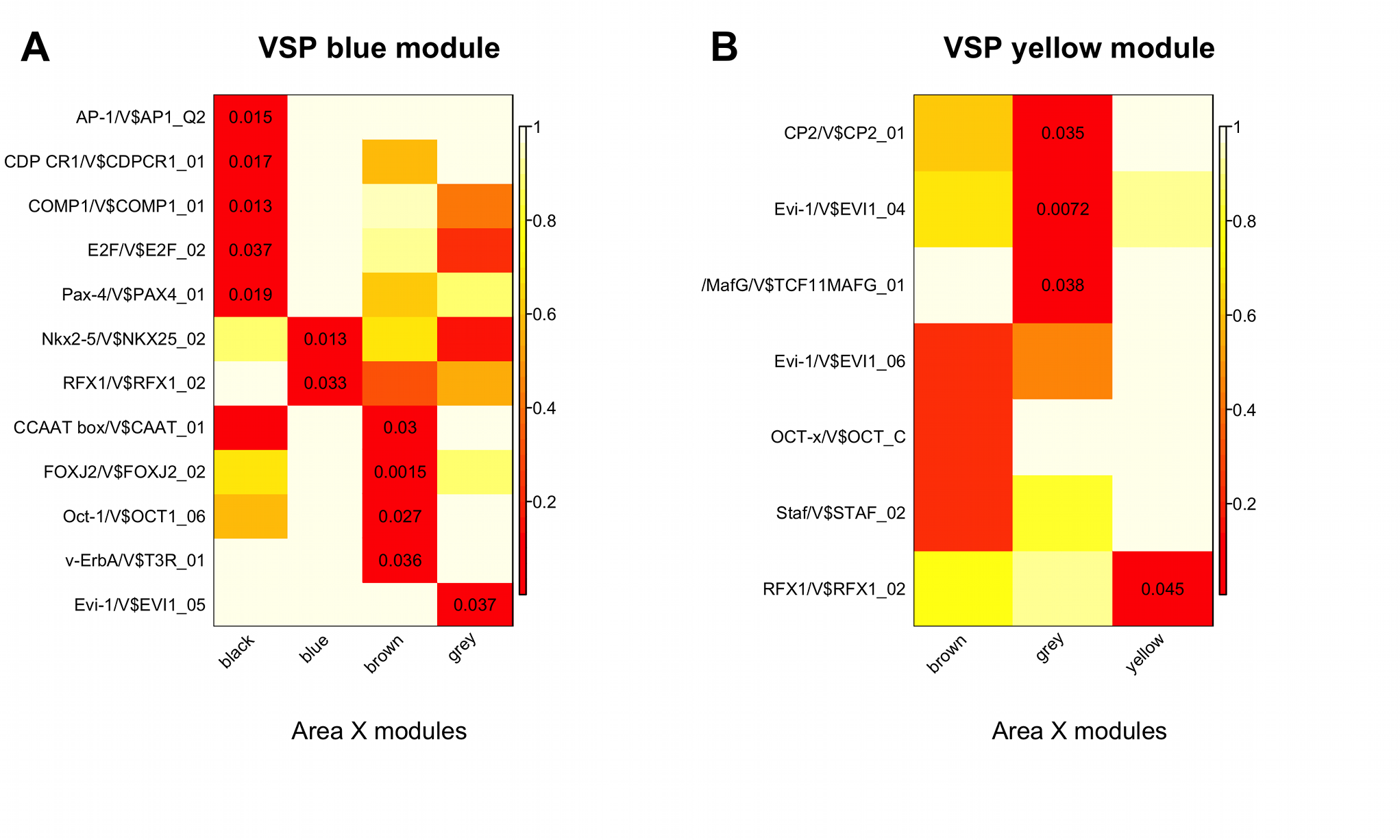

Supplement: Figure S4 — Enrichment of specific transcription factor binding sequences (TFBSs) in functionally distinct subsets of VSP-specific modules. Heatmaps of enrichment p-values for TFBSs (rows) in subsets of the genes from the VSP-specific blue (A) and yellow (B) modules, segregated by their area X module assignments (columns). Previous analysis of results from DAVID found that, based on their distribution in area X modules, subsets of the VSP blue and yellow modules were enriched for distinct sets of biological functions. Actual p-values are shown in cells with p<0.05, as determined via Fisher's exact test by comparison to TFBSs over-represented in the module as a whole. An important caveat is that these results were based on TFBSs and upstream sequences from the human genome, since PAINT (Promoter Analysis and Interaction Network Tool, www.dbi.tju.edu/dbi/tools/paint; Vadigepalli et al., 2003) is currently limited to human, mouse, and rat data. Multiple lines of evidence suggest that the neural systems supporting learned vocalization are highly analogous in humans and zebra finches (Jarvis, 2004), thus these sequences may or may not be the same in birds. In light of this, the enrichment for the FOXJ2_02 transcription factor in the area X brown module, which is highly singing-related (see Figure S3), is potentially interesting since its core binding sequence is very similar to that of FOXP2 (Pérez-Sánchez et al., 2000; Spiteri et al., 2007), a well known singing-related transcription factor. (TIFF) [file pcbi.1002773.s004.tif]
