# Supplementary material for: Distinct Neurogenomic States in Basal Ganglia Subregions Relate Differently to Singing Behavior in Songbirds
Source: PLoS Comput Biol. 2012 Nov 8;8(11):e1002773. doi: 10.1371/journal.pcbi.1002773 (PMC3493463; doi:10.1371/journal.pcbi.1002773)
Supplement: Text S1 — References for supporting information. (DOC) [file pcbi.1002773.s008.doc]

**References for supporting information**

Hilliard AT, Miller JE, Fraley E, Horvath S, White SA (2012) Molecular microcircuitry underlies functional specification within a basal ganglia circuit dedicated to vocal learning. Neuron 73: 537-52.

Jarvis ED (2004) Learned birdsong and the neurobiology of human language. Ann. NY Acad. Sci. 1016: 749-777.

Langfelder P, Horvath S (2008) WGCNA: an R package for weighted correlation 35network analysis. BMC Bioinformatics 9: 559.

Langfelder P, Luo R, Oldham MC, Horvath S (2011) Is my network module preserved and reproducible? PloS Comp. Biol. 7: e1001057.

Spiteri E, Konopka G, Coppola G, Bomar J, Oldham M, et al. (2007) Identification of the transcriptional targets of FOXP2, a gene linked to speech and language, in developing human brain. Am. J. Hum. Genet. 81: 1144-57.

Vadigepalli R, Chakravarthula P, Zak DE, Schwaber JS, Gonye GE (2003) PAINT: a promoter analysis and interaction network generation tool for gene regulatory network identification. OMICS. 7: 235-52.

Pérez-Sánchez C, Gómez-Ferrería MA, de La Fuente CA, Granadino B, Velasco G, et al. (2000) FHX, a novel fork head factor with a dual DNA binding specificity. J. Biol. Chem. 275: 12909-16.
